# Evaluation of Axial Preload in Different-Frequency Smart Bolts by Laser Ultrasound

**DOI:** 10.3390/s22228665

**Published:** 2022-11-10

**Authors:** Guanpin Ren, Huan Zhan, Ziqian Liu, Wei Jiang, Ru Li, Shuang Liu

**Affiliations:** 1 Department of Applied Physics, College of Mathematics and Physics, Chengdu University of Technology, Chengdu 610059, China; 2 Chengdu Development Center of Science and Technology of CAEP, Chengdu 610299, China; 3 School of Big Data and Artificial Intelligence, Chengdu Technological University, Chengdu 611730, China

**Keywords:** laser inspection system, bolt preload, intelligent sensor, ultrasonic TOF, non-destructive testing

## Abstract

We report here on a laser ultrasonic system to indirectly evaluate the preload force of different-frequency piezoelectric bolts. This newly developed system enables us to achieve the goal of non-contact excitation and synchronously collects the laser-induced ultrasonic signal by the combination of a smart piezoelectric sensor and a magnetically mounted transducer connector. A numerical model based on the finite element method (FEM) was developed to simulate the propagation and displacement distribution of laser-generated ultrasonic waves along the axial direction. The measured A-scan waveform basically coincided with the counterpart obtained from a theoretical simulation, confirming the effectiveness of the proposed system to measure a bolt. By comparison, a laser spot diameter of 6 mm was the optimal beam diameter for the excitation of the ultrasonic wave in the bolt. The linear relationship between time of flight (TOF) of the ultrasonic longitudinal wave and bolt torque was almost independent from the center frequency of the smart bolt. By contrast, a piezoelectric patch centered at 5 MHz was more suitable as an ultrasonic sensor in terms of the nonlinear effects component suppression and linear fitting degree between TOF and torque. The results indicate that the proposed system based on a surface-mounted piezoelectric sensor is a promising system for evaluating the axial preload change of connector and fastener and is an additional potential laser ultrasonic system for nondestructive tests.

## 1. Introduction

Bolts are important fasteners indispensable in the manufacturing and national defense industries and are critical components of important infrastructure, such as aerospace, vehicle, ship, railway, bridge, and building facilities [1,2]. As the use of bolt increases, there is a greater need for the accurate control of bolt preload during tightening and in situ online monitoring or the evaluation of the residual axial force during the service life of the connection. Insufficient preload will cause vibration, slack, and slipping of structural connections, which can damage the integrity of the structure and affect the normal operation of any bolt-assembled equipment. Instead, an excessive preload will cause a severe plastic deformation of the bolt, which could lead to failure due to fatigue or fracture [3,4]. On this basis, researchers and engineers around the world are striving to develop on-line reliable methods to evaluate and measure the bolt preload force. These methods can be roughly divided into three categories: percussion detection, vision detection and sensor detection. It is difficult to achieve high precision using percussion and vision detection because of variations in the actual working conditions, low accuracy of the construction equipment and lack of experience among workers [5,6,7]. Other sensors, such as the strain sensor and the fiber Bragg grating (FBG) sensor, have also been developed in recent years and have been proven to evaluate the bolt preload force [8,9,10,11]. However, most of the sensors have different intrinsic shortcomings and are found unsuitable to evaluate or measure the bolt preload force [8,9,10,11]. For instance, conventional strain gauges cannot adhere to fully threaded bolts, and the FBG sensor embedded into the bolt body measures the preload force by the relative displacement of Bragg Wavelength. It is of no use for small bolts and causes damage to the bolt structure. In addition, its performance is strongly attenuated at high temperature, thus this sensor is not suitable for high-temperature environments.

In contrast to the above methods, benefiting from their advantages of high precision, excellent real−time performance, and strong sensing penetration, ultrasonic sensors have been widely used for the detection of axial stress in bolts since the 1980s [12,13,14,15]. A great deal of research has been conducted on ultrasonic theory and equipment to determine or evaluate the axial preload in bolts [16,17,18,19,20,21,22,23,24]. Although piezoelectric transducers are widely used, the accuracy of ultrasonic detection is relatively low due to poor bolt surface conditions and unstable coupling properties. During measurement, the ultrasonic wave passes through the coupling layer twice. As a result, unevenness and inconsistency of the coupling layer can produce errors of the same order of magnitude as the measured value. In order to improve the coupling performance, an electromagnetic acoustic transducer (EMAT) and a permanent mounted transducer system (PMTS) were introduced, gradually replacing the piezoelectric transducer in recent years. Nevertheless, EMAT has obvious drawbacks, such as low conversion efficiency and difficulty in exciting the longitudinal wave on the end faces of ferromagnetic bolts. Currently, the widespread application of PMAT is also limited by special handling processes of the bolt.

In addition to the previous methods, a few researchers [25,26,27] apply laser ultrasonic systems to monitor and estimate the looseness in bolted joints. For example, based on the Hertzian theory of contact, Yang Zhou et al. [27] applied laser-induced wave energy dissipation to monitor the looseness of a bolt. Ju−Won Kim et al. [26] reported the automatic estimation of looseness in a bolt joints by the combination of a non-contact laser scanning system and a deep convolutional neural network (DCNN). Studies in the literature applied a laser−induced guide wave rather than a single longitudinal wave (a longitudinal wave is more sensitive to preload changes) [12,13] to monitor and estimate the looseness of a bolt. Because there are few reports on the measurement of the bolt preload force by using a laser as a signal excitation source, all studies have used the Hertz contact method with this excitation mode, and the area of laser action was the surface of the connected parts; by analyzing the ultrasonic attenuation signal on the other side, the looseness of bolts was evaluated. In this work, a nanosecond pulsed laser used as a pump source directly irradiated the bottom of the bolt. The laser thermal effect area on the bolt itself, strictly in line with the thermoelastic effect, did not cause damage to the bolt, in line with non−destructive testing requirements, and the bolt head received the single longitudinal wave signals through a smart piezoelectric sensor and a magnetically mounted transducer connector. A numerical simulation of the longitudinal wave in the bolt induced by a nanosecond laser pulse was carried out. The influence of the laser spot diameter on a single longitudinal wave signal was investigated. By the TOF change, the axial preload change of the smart bolt could be evaluated effectively. The 5 MHz piezoelectric sensor showed advantages over the 2 MHz and 10 MHz piezoelectric sensors in terms of nonlinear effects and linear fitting degree between the TOF and the torque.

## 2. Research Methods

### 2.1. Measurement Principle of Bolt Preload

**Figure 1:**
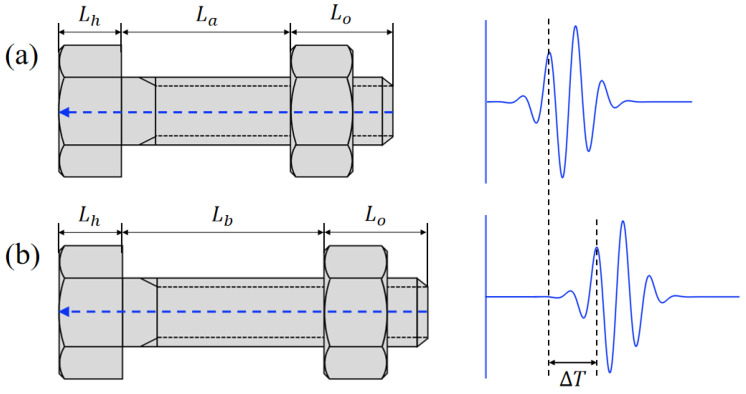
Fundamental principle to measure the bolt preload force by the mono-wave method: (**a**) TOF of the pulse−echo before tightening; (**b**) TOF of the pulse−echo after tightening.

The preload force measured by an ultrasonic sensor is based on the acoustic−elastic effect. The mono−wave method, i.e., the use of a single transverse wave or longitudinal wave, is widely used to measure the axial stress in a bolt. The longitudinal wave is frequently used in engineering applications because it is more sensitive to changes in stress or preload force. Figure 1 shows the basic principle of the mono-wave method. The preload force can be calculated by measuring the TOFs of the pulse−echo before and after tightening. The relationship between TOF and preload force can be expressed as follows:

(1) $$F=\frac{E\cdot S\cdot \Delta L}{L}$$

where F is the preload force, ΔL=ΔT·V is the change of bolt length, ΔT is the change in TOF from before to after tightening, and V is the propagation speed of the ultrasonic longitudinal wave in the material; E is the elastic modulus of the bolt material, S is the effective cross−sectional area, and L is the clamping length of the bolt. In this relation, the TOF of the ultrasonic wave is positively correlated with the preload force. It is well known that different batch bolts correspond to different elastic moduli E and therefore to the absolute preload values. Considering this, we only measured the TOF of the first longitudinal wave to evaluate the bolt axial preload in this work. The whole experiment was performed in a temperature−controlled laboratory. Thus, the influence of the temperature on the velocity of the longitudinal wave and the elastic modulus of the bolt material was not considered here to simplify the process.

### 2.2. Laser Ultrasonic Measurement System

**Figure 2:**
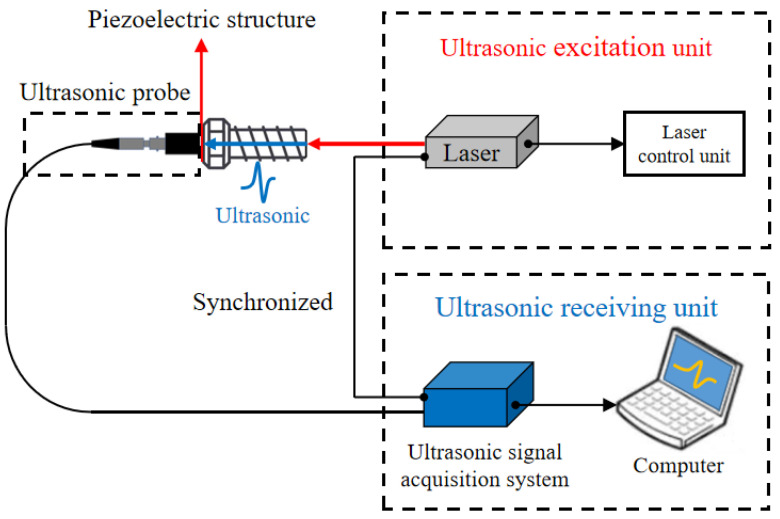
Schematic diagram of the laser ultrasonic system for bolt preload force evaluation.

The excitation source of the ultrasonic wave was a commercial nanosecond pulsed laser at 1064 nm in this work. The collimation laser beam had a spot diameter of 8 mm and a pulse width of 10 ns. The repetition rate could be adjusted from 1 Hz to 20 Hz. The laser output energy was 30 mJ, and the energy stability (RMS) was less than 1%. The independently developed instrument could collect ultrasonic wave signals at a frequency from 0.2 MHz to 25 MHz. The corresponding sampling frequency was 100 MHz, and the maximum gain value was as high as 89 dB. A schematic diagram of the laser ultrasonic measurement principle is presented in Figure 2. The laser beam pulse directly irradiated the bottom of the bolt, and then induced an ultrasonic longitudinal wave which propagated along the axial direction of the bolt through the thermo-elastic effect. Simultaneously, a smart piezoelectric sensor received an ultrasonic signal, and a magnetically mounted transducer connector converted the ultrasonic signal covert into a voltage signal above 100 mV. To ensure the effectiveness and reliability of the collected signal, the use of a BNC connector line achieved synchronization between the laser excitation unit and the signal receiver unit.

**Figure 3:**
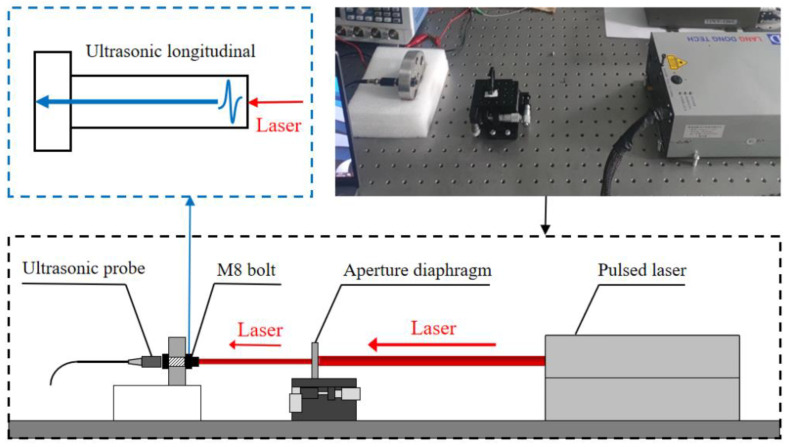
Schematic diagram and experimental setup of the laser ultrasonic excitation unit.

**Figure 4:**
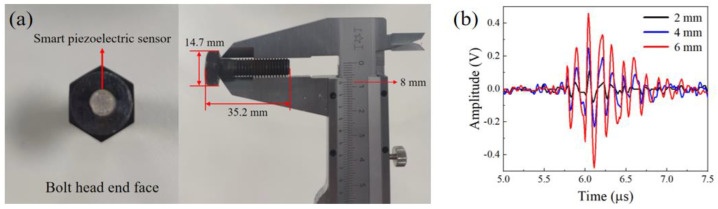
(**a**) M8 bolt with a smart piezoelectric sensor; (**b**) ultrasonic signal strength for different spot diameters.

The optical path of the laser excitation unit is shown in Figure 3. The ultrasonic probe was the developed magnetically mounted transducer connector. Through a commercial laser cleaning and polishing system, the surface roughness of the bolt head was controlled below 1 μm. Three different−frequency smart piezoelectric sensors were directly bonded on the surface of the bolt head by epoxies such as Loctite ABLESTIK 104, as a viscous coupling layer. After baking–curing in a low-temperature furnace for 2 h, a viscous couplant layer formed. The corresponding center frequencies were 2 MHz, 5 MHz, and 10 MHz, respectively. After passing through the aperture diaphragm, the collimated laser vertically irradiated the bottom of the bolt. Subsequently, an ultrasonic wave was induced through a thermo-elastic effect on the surface [28]. The induced signal component was mainly the ultrasonic longitudinal wave propagating from the bottom to the bolt head. The measured bolt in this work was an M8 carbon steel bolt with a nominal diameter of 8 mm and a length of 35.2 mm (Figure 4a). Considering that the laser irradiation area is the solid region free from thread, an aperture diaphragm was used to adjust the laser spot diameter from 2 mm to 6 mm. The influence of the laser spot diameter on the ultrasonic wave signal was investigated and is shown in Figure 4b. By comparison, the spot diameter of 6 mm corresponded to a higher signal amplitude and better signal quality. Therefore, the 6 mm diameter laser beam was the optimal ultrasonic wave excitation source in this work.

## 3. Ultrasonic Measurement of the Bolt

### 3.1. Theoretical Model and Simulation

Laser energy absorption occurs on the surface and sub-surface of a bolt, regarded as the boundary heat source. The energy expression is

(2) Q=I·f(x)·g(t)

where Q is the heat source, I is the energy absorbed by the material, and f(x) and g(t) are the laser spatial distribution function and the time distribution function, respectively. The laser source beam is the Gaussian beam, and its distribution function can be expressed as

(3) $$\{\begin{array}{c} f\left(x\right)=exp\left(-\frac{x^{2}}{r_{0}^{2}}\right)\\ g\left(t\right)=\frac{t}{t_{0}}exp\left(-\frac{t}{t_{0}}\right) \end{array}\;$$

where $r_{0}$ is the laser spot radius, and $t_{0}$ is the laser pulse width.

**Figure 5:**
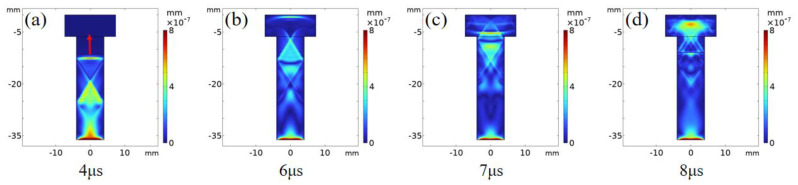
Displacement of the mechanical vibration caused by thermo−elastic effects in the bolt; dissemination process: (**a**)→(**b**)→(**c**)→(**d**).

**Figure 6:**
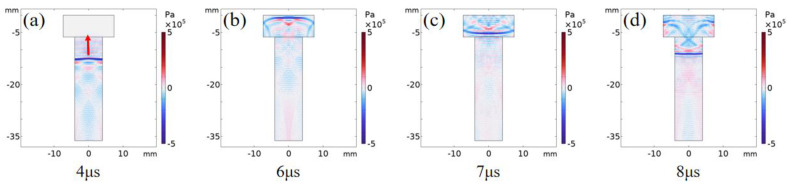
Ultrasonic longitudinal wave propagating along the axial direction in the bolt; dissemination process: (**a**)→(**b**)→(**c**)→(**d**).

The finite element method [29,30] is used to simulate the propagation process of laser ultrasound inside the bolt. Figure 5 shows the displacement distribution of the ultrasonic wave from 4 μs to 8 μs. It is important to note that the main vibration energy was transmitted along the axial direction of the bolt and was reflected on both sides of the bolt to generate the trailing wave (Figure 5a). At 6 μs, the main vibration reached the boundary of the bolt head (Figure 5b) and was then reflected (Figure 5c). Part of the reflection was transmitted along the opposite axial direction, and the remaining wave secondary reflection occurred at the internal boundary of the bolt head (Figure 5d). Figure 6 shows the propagation process of the ultrasonic wave between 4 μs to 8 μs. The wave front propagated along the bolt axis (Figure 6a), reached the bolt head boundary at 6 μs (Figure 6b), and was reflected at the bolt head (Figure 6c). Some reflected waves appeared in the secondary reflection at the bolt head structural boundary (Figure 6d). Since the ultrasonic wave was generated by mechanical vibration displacement, the propagation process in Figure 5 and Figure 6 is consistent.

### 3.2. Ultrasonic Signal Acquisition

**Figure 7:**
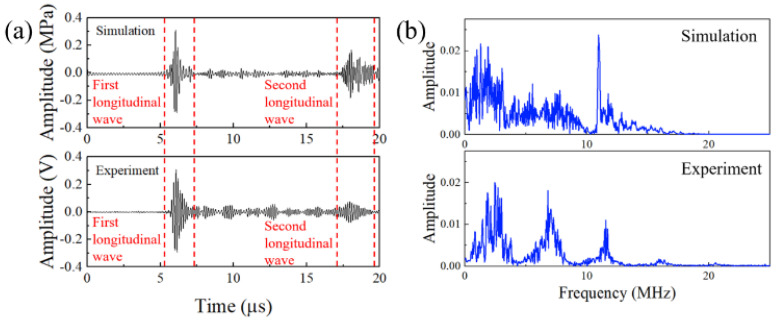
(**a**) Ultrasonic signals obtained from the simulation and the experiment; (**b**) FFT spectrum of the simulation and experiment’s ultrasonic signal.

To examine the effectiveness of the laser ultrasonic system for bolt preload evaluation, the simulated A−scan waveform and measured A−scan waveform from the ultrasonic acquisition unit were compared, as shown in Figure 7a. We noted that the profile curve of the simulated A−scan waveform was almost in accordance with that of the measured one. The first longitudinal wave signal envelope can be easily observed in Figure 7 and originated from the interaction between the laser-induced ultrasonic wave and the head boundary. After propagating three times along the axial direction, the bolt head could detect the second longitudinal wave. The multiple small peaks between the first and the second longitudinal wave correspond to trail waves and reflection waves from the bolt head region. The amplitude intensity of the second longitudinal wave presented a reduction compared with its counterpart in the simulation results. This was due to the bolt intrinsic loss caused by dissipation, absorption and scattering and the slight deviation of the wave transmission direction caused by the roughness and uncleanness of the bolt surface. Thus, the first longitudinal wave was more suitable for the measurement of the bolt preload force in our work.

**Figure 8:**
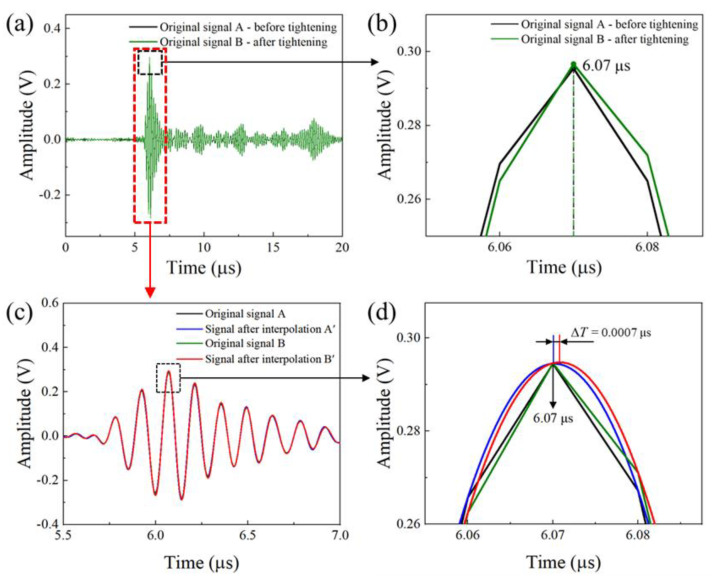
(**a**) Original signal before and after applying a small preload; (**b**) the waveform underwent a slight phase shift, but the data of the sampling points at the peak remained the same; (**c**) original sample data (temporal signals A, B) and interpolation waveforms (interpolated signals A’, B’) before and after loading; (**d**) rectangular window for comparing the TOF at the peak position between pre- and post−interpolation.

Limited by the 100 MHz of the available data acquisition frequency, the time accuracy was relatively low. In our experiment, the TOFs of the pulse−echo presented no change when the axial preload force was below 1 kN, as shown in Figure 8a,b; although the signal waveform showed a slight phase shift, the TOF of the peak point was still the same, because of the original sampling frequency. To address this issue, we applied the spline function interpolation [31] to increase the data points and improve the resolution of the propagation time difference. We inserted 100 interpolation points between the two original time data points. The time interval was improved from 10 ns to 100 ps. The original temporal signal A and B correspond to the longitudinal wave signal before and after a slight tightening, as shown in Figure 8a. We found no change of TOF between signal A and signal B. The interpolation algorithm made the signal curves A and B convert into the corresponding curves A’ and B’. The curve profiles became smooth, as indicated in Figure 8b. With the introduction of the interpolation algorithm, a small TOF change was observed at the signal peak position, and the corresponding change value was 0.7 ns (see Figure 8b).

**Figure 9:**
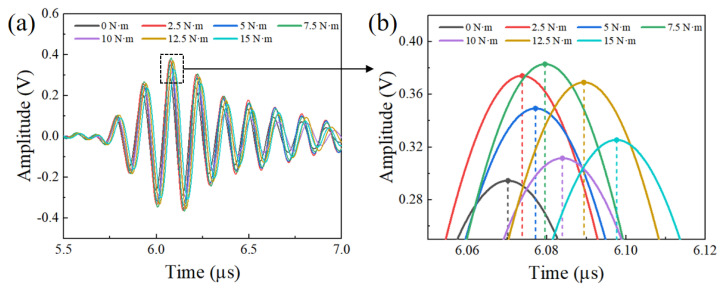
(**a**) Received signals from the ultrasonic sensor during different torque−tightening processes; (**b**) rectangular window demonstrating the TOF change before and after different torque tightening processes.

The preload force of the bolt of this work was inconvenient for a commercial tensile machine. A manual torque wrench was used here to indirectly control the bolt preload through a tightening torque, although only about 10% of the torque was converted into axial preload. A bolt was gradually installed by a torque wrench with a torque from 0 N∙m to 15 N∙m, and the preload increase interval was designed to be 2.5 N∙m. For each torque, the laser ultrasonic system improved the measurement precision by averaging the testing ultrasonic signal of measurements repeated 20 times. In addition, time−domain averaging and an FIR filter were adopted to suppress noise interference. In order to investigate the relationship between the torque and the TOF change, the ultrasonic signals during different torque tightening processes were received by a smart sensor, as shown in Figure 9a. With the increase of the torque, the highest peak position of the first longitudinal wave presented the time−decay phenomenon. The ultrasonic signal amplitude had a small range of fluctuations, as indicated in Figure 9b. This could be attributed to a slight variation of the laser beam irradiation position and the small noise inference of the acquisition unit.

For different torques, the bolt was subjected to different forces, which made the bolt elongation change. The time variation of signal T (TOF) is the key data we use to evaluate a preload. It directly reflects the elongation change of the bolt force, but the amplitude of the signal is not the value we pay attention to. In the experiment, we also found that due to the small interference of the measuring equipment and external signals, the amplitude of the signal constantly fluctuated up and down, but this vibration did not affect our acquisition of the TOF and the overall measurement results. Figure 9 shows one set of data from all repeated experiments. For each set of different data, the amplitude of the signal was also different. However, the phase shift (TOF) on the time axis was strictly in line with physical laws and expectations.

**Figure 10:**
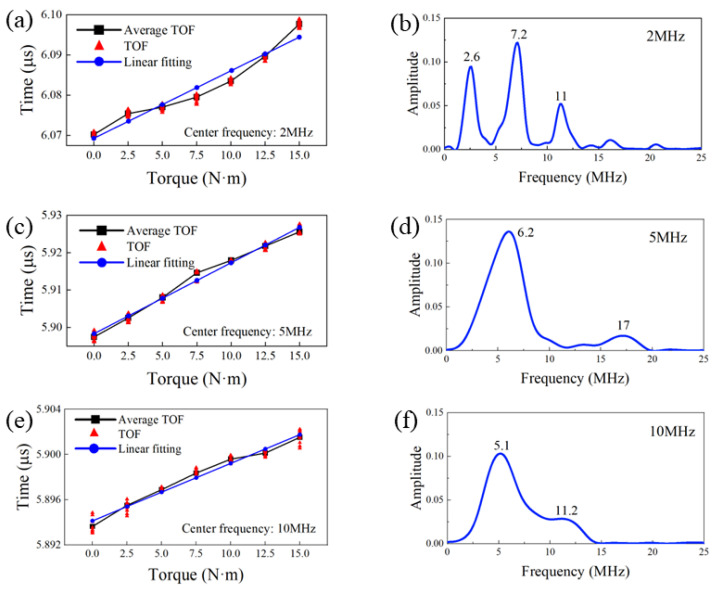
Relationship between the TOF and the preload torque for different center frequency sensors: (**a**) 2 MHz, (**c**) 5 MHz, (**e**) 10 MHz; spectrum of the first longitudinal wave signal for (**b**) 2 MHz, (**d**) 5 MHz, and (**f**) 10 MHz PZT sensors.

For the increase in the amount of phase shift from 12.5 N∙m to 15 N∙m, as shown in Figure 10a, the relationship was not strictly linear. This was true only for bolts with 2 MHz sensors, as the linearity of 5 MHz and 10 MHz bolts was obviously better. However, this did not affect the validity of the preload measurement, which, we think, was affected by the sensor itself.

The TOF of the laser-induced longitudinal wave as a function of the torque for three different-frequency smart bolts was measured and is shown in Figure 10a,c,e. With the increase of the preload applied to the bolts, the TOF of the longitudinal wave presented a nearly linear increase, demonstrating an extension of the axial length of the bolts. Nonlinear ultrasonic effects were easily induced due to the special bolt shape, heterogeneous extension of the length, and intense ultrasonic amplitude induced by the laser. On this basis, frequency spectra of the three different sensor bolts were obtained by the Fastest Fourier Transform in the West (FFTW) algorithm. The corresponding results are presented in Figure 10b,d,f. It is important to note that all the three different sensor bolts presented broad frequency spectra. This was due to a wide−band ultrasonic signal induced by the pulsed laser. In addition to this, obvious harmonic waves induced by nonlinear effects can be easily observed. As shown in Figure 10b, the center frequency of the 2 MHz smart bolt was at 2.6 MHz, and the components of the third and four harmonic generations can be clearly seen. The deviation of the frequency center value could be caused by the piezoelectric material and frequency shift induced by the special bolt structure. In addition, no second harmonic generation component was found. This was probably related to the intrinsic property of the piezoelectric material. The frequency spectrum of the 5 MHz smart bolt also presented no second harmonic generation component. The intensity of the higher harmonic generations was weaker than the center frequency intensity and counterpart of the 2 MHz smart bolt. For the 10 MHz smart bolt, the center frequency peaked at 11.2 MHz. No higher harmonic generation component was observed. However, a strong low−frequency response centered at 5.2 MHz could be observed. The reason is still unclear and will be further explored in the near future. According to the nonlinear effects component proportion and the linearly fitting degree between the TOF and the torque, the 5 MHz piezoelectric sensor appeared more suitable as an ultrasonic sensor for the evaluation of the axial preload.

**Figure 11:**
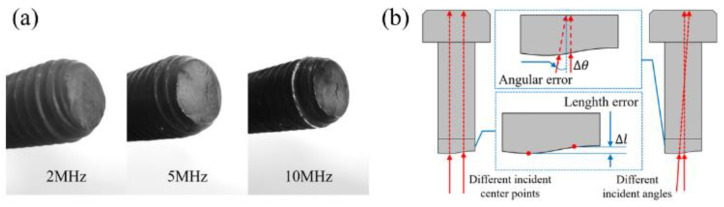
(**a**) Photograph of the bottom−end faces of three intelligent bolts; (**b**) errors caused by incident center and incident angle offset.

To further analyze the deviation between the actual measurement and the linearly fitted result, a photograph of the bottom end face of the smart bolts was taken, as shown in Figure 11a. The bottom of the bolt presented an obvious, uneven distribution. A two dimensional (2D) cross−sectional snapshot of the bolt is presented in Figure 11b. The deviation was mainly induced by the uneven bottom of the bolt, the length error Δl caused by the offset of the laser incidence center position during multiple tests, and the angular error Δθ of the incident position originated from the incomplete collimation of optical path and the tilted bottom surface of the bolt. The slight loosening of the bolts during testing caused by friction failure of the bolt die also affected the stress stability of the bolts. Additionally, signal interference of the laser excitation source and electronic noise of the ultrasonic acquisition unit contributed to the deviation.

The pure laser−based ultrasonic technique, i.e., the laser generation−laser receiving technique, has been intensely investigated recently in various industrial fields. Nevertheless, for strongly scattering surfaces, high−powered detection lasers are needed as well as complex interferometer set−ups, which may be costly and difficult to miniaturize. The laser ultrasonic system proposed here combines the advantages of laser non−contact excitation and good−stability ultrasonic sensing free from a couplant. In addition to this, the combination of a smart piezoelectric sensor and a magnetically mounted transducer connector allows for much higher sensitivity with a lower cost in ultrasound detection than laser interferometers. In this work, we applied this proposed system to successfully measure the TOF of a laser ultrasonic longitudinal wave and indirectly evaluate the relationship between the bolt preload and the TOF of the ultrasonic wave. This work indicates that this system can be used as another effective laser ultrasonic method for non−destructive testing (NDT) applications in the near future.

## 4. Conclusions

In this paper, we successfully measured the TOF of an ultrasonic longitudinal wave by applying a pulsed laser as the ultrasonic excitation in combination with a newly developed ultrasonic sensor (i.e., a smart piezoelectric sensor and a magnetically mounted transducer connector). The propagation process and displacement distribution of the laser-induced ultrasonic wave were fully demonstrated along the axial direction of the bolt. The experimental testing results agreed very well with the phenomena observed in the simulation results. The introduction of interpolation made the TOF change measure of the ultrasonic longitudinal wave accurate and effective. The TOF change of the first longitudinal wave presented an almost linear increase with the increase of the torque. Compared with the piezoelectric patches centered at 2 MHz and 10 MHz, the piezoelectric patch centered at 5 MHz showed a better suppression ability of more intense nonlinear effects and a better degree of linear fitting between the TOF and the torque. Future research work will focus on the optimization of the laser ultrasonic measurement system to reduce external errors, the analysis and verification of nonlinear ultrasonic effects of the piezoelectric sensor at a microscopic level, and the combination with deep learning algorithms for axial preload evaluation.
